# 非小细胞肺癌术后辅助治疗中国胸外科专家共识（2018版）

**DOI:** 10.3779/j.issn.1009-3419.2018.10.01

**Published:** 2018-10-20

**Authors:** 非小细胞肺癌辅助治疗胸外科共识专家组

## 前言

肺癌对人类健康的威胁日益严重，2012年全世界有182.5万新发病例，158.9万因肺癌相关死亡^[1]^。据最新统计资料显示，2014年我国肺癌新发病例78.2万，死亡病例62.6万。在发病率方面，肺癌在男性中居第一位，女性中居第二位，仅次于乳腺癌。而死亡率无论在男性还是女性中均居第一位^[2]^。非小细胞肺癌（non-small cell lung cancer, NSCLC）是肺癌中最常见的一种类型，约占肺癌的80%-85%。对于早期NSCLC，完全手术切除仍然是提高患者生存率的关键。此外，对于局部晚期NSCLC，尤其是可手术切除的Ⅲa期患者，完全性手术切除联合术后辅助化疗仍然是治疗的主要模式。作为肺癌多学科综合治疗的一种重要模式，术后辅助治疗是外科根治性切除的重要组成部分。

为此，中国抗癌协会肺癌专业委员会外科学组成员、EVAN研究的16家研究中心专家、其他中国著名的拥有肺癌辅助治疗经验的胸外科专家撰写“非小细胞肺癌术后辅助治疗中国胸外科专家共识”（简称“共识”），以期推动我国术后辅助治疗药物的合理使用，进一步提高我国肺癌规范化诊疗水平。本共识将重点放在作为NSCLC两种主要亚型的腺癌和鳞癌的术后辅助治疗上，重点回答以下3个问题；问题一：Ⅰ期-Ⅲa期行完全手术切除的NSCLC，哪些患者需要行术后辅助治疗？问题二：Ⅰ期-Ⅲa期行完全手术切除的NSCLC，若需要术后辅助治疗，需要何种类型的药物？问题三：Ⅰ期-Ⅲa期行完全手术切除的NSCLC，哪些患者需要行术后辅助放疗。

## 方法学

执笔专家依据现有数据库（检索方法如下描述）和临床经验，整理包含辅助治疗关键临床问题的文章初稿，于共识启动会与核心专家组成员进行讨论。专家组对于本次使用的推荐级别定义达成一致，并对临床治疗意见形成初步共识。为最大化降低共识委员会和执笔专家的意见偏倚，进一步汇集近100位其他中国著名的拥有良好肺癌术后辅助治疗经验的胸外科专家意见，最终由核心专家组定稿会讨论定稿。

通过EMBASE和PubMed两个数据库进行检索，检索截止时间为2018年5月4日，应用与NSCLC化疗、放疗、靶向治疗、免疫治疗和抗血管生存治疗相关的关键词和医学主题词进行检索，完整检索式如下。

通过检索式'non small cell lung cancer'/exp AND ('adjuvant therapy'/exp OR 'postoperative care'/exp)在EMBASE数据库中进行检索，共得到6, 723篇文献。

通过检索式('carcinoma'[title/abstract] AND 'non-small-cell'[title/abstract] AND 'lung'[title/abstract]) OR 'non-small-cell lung carcinoma'[title/abstract] OR 'NSCLC'[title/abstract]) AND ('antineoplastic combined chemotherapy protocols/therapeutic use'[MeSH Major Topic] OR 'carcinoma, non-small-cell lung/drug therapy'[MeSH Major Topic] OR 'carcinoma, non-small-cell lung/radiotherapy'[MeSH Major Topic] OR 'carcinoma, non-small-cell lung/therapy'[MeSH Major Topic] AND ((Clinical Trial, Phase Ⅲ[publication type] OR Review[publication type] OR Meta-Analysis[publication type] OR Clinical Trial[publication type])”在PubMed数据库中进行检索，共得到9, 835篇文献。经过Endnote去掉两个数据库中重复的文献，共得到15, 515篇。

通过检索式“（摘要：辅助化疗或摘要：辅助放疗或摘要：辅助靶向治疗）和（标题：非小细胞肺癌）”分别在万方医学数据库和中国知网中进行检索并去重，共得到126篇文献。

所有推荐基于非小细胞肺癌第八版新分期（表 1）^[3]^。

**1:** 推荐级别

级别	定义
1A	基于高水平证据，专家组统一共识，强烈推荐
1B	基于高水平证据，专家组小有争议，积极推荐
2A	基于中等水平证据，专家组统一共识，推荐
2B	基于中等水平证据，专家组小有争议，一般推荐
3	基于低水平证据，专家组存在争议，不推荐

## 问题一：Ⅰ期-Ⅲa期行完全手术切除的NSCLC，哪些患者需要行术后辅助治疗？

**推荐1：Ⅰ期，不需要辅助化疗；1A**

到目前为止，几乎所有的NSCLC术后辅助化疗研究，均不包括Ⅰa期（第七版分期，肿瘤大小 < 3 cm，N0）患者。2008年NSCLC顺铂辅助协作组（Lung Adjuvant Cisplatin Evaluation Collaborative Group, LACECG）的荟萃分析包括了IALT^[4]^、JB10^[5]^、ANITA^[6]^、ALPI^[7]^和BLT^[8]^在内的5个含铂化疗方案的随机研究^[9]^，共纳入4, 584例NSCLC患者，化疗组Ⅰa期NSCLC患者185例，观察组Ⅰa期NSCLC患者162例，根据分期进行亚组分析发现，Ⅰa期患者化疗组与观察组比较，在总体生存期指标上并不能获益，危险比（hazard ratio, HR）为1.40 [95%可信区间（CI）：0.95-2.06]。这为完全切除术后的Ⅰa期NSCLC不需辅助化疗提供了证据。

美国肿瘤和白血病B组的CALGB9633临床试验是第一个针对Ⅰb期NSCLC患者的临床试验，该试验共纳入344例Ⅰb期NSCLC患者，随机分为4个周期的辅助化疗和对照组，化疗组泰素200 mg/m^2^，卡铂AUC=6，21 d为1个周期，共4个周期，其中86%的患者完成了4个周期的化疗。中位随访74个月时，两组生存没有显著差异（HR=0.83, 90%CI: 0.64-1.08, *P*=0.12）。特别是对于肿瘤 < 4 cm的患者，辅助化疗并不能改善生存（HR=1.12，90%CI: 0.75-1.07, *P*=0.32）^[10]^。这是第一个采用第3代化疗方案作为Ⅰb期NSCLC术后辅助化疗有统计学意义的研究报道。2005年发表在《新英格兰医学杂志》上的加拿大国立癌症研究院的JBR10试验是第一项对入组患者完全采用第3代化疗方案的临床试验，该试验纳入了482例Ⅰb期（T2N0）或Ⅱ期（T1N1或T2N1）NSCLC，随机分为化疗组和对照组，化疗组：顺铂50 mg/m^2^ d1、d8，28 d为1个周期，共4个周期，长春瑞滨25 mg/m^2^ d1，7 d为1个周期，共16个周期。针对Ⅰb期NSCLC患者进行分析显示，观察组和术后辅助化疗组的总生存率并无显著统计学差异（*P*=0.79）^[5]^。2008年LACECG的荟萃分析发现，Ⅰb期患者化疗组与对照组比较，在总体生存期指标上并不能获益，HR为0.93（95%CI: 0.78-1.10）^[9]^。因此，现有证据均提示对于Ⅰ期NSCLC患者，不推荐进行辅助化疗。

**推荐2：Ⅱa期，不常规推荐辅助化疗（术后综合评估包括与肿瘤内科专家会诊，评估辅助化疗对于每个病人的效益和风险，在做出建议时，还要考虑肿瘤分期以外的其他因素，包括组织病理学特征和基因改变等。当有证据支持，专家组有统一认识，利大于弊，可考虑给予辅助化疗）；2B**

术后辅助化疗对肿瘤大小4 cm-5 cm（N0M0）的患者获益情况来源于特定的亚组分析。CALGB9633临床研究中位随访时间74个月时，探索性分析肿瘤大小≥4.0 cm的患者发现，与对照组相比，化疗组死亡率降低31%（HR=0.69, 90%CI: 0.48-0.99, *P*=0.043），化疗组无病生存率提高31%（HR=0.69, 90%CI: 0.49-0.97, *P*=0.035）^[10]^。中位随访时间为9.3年时，再一次分析肿瘤大小≥4.0 cm的患者发现，与对照组比较，化疗组有获益趋势但未达到统计学差异（HR=0.77, 90%CI: 0.57-1.04, *P*=0.079）^[11]^。JBR10试验长期随访结果于2010年发表在《临床肿瘤学》杂志上，当中位随访时间为9.3年，探索性分析肿瘤大小≥4.0 cm的患者发现，与对照组相比，化疗组总生存率并未显著改善（HR=0.66, 95%CI: 0.39-1.14, *P*=0.13）^[12]^。这两项临床试验的亚组分析提示我们，术后辅助化疗可能提高肿瘤大小≥4.0 cm患者（Ⅱa期）的总生存率。然而，不确定性依然存在，因为长期随访结果并未达到统计学差异，可能是样本量较小或者是较宽的可信区间导致的。总之，对于Ⅱa期NSCLC（肿瘤直径肿瘤直径＞4 cm，≤5 cm，N0M0），术后辅助化疗并不能提高长期生存率。

**推荐3：Ⅱb期-Ⅲa期，常规辅助化疗；1A**

1995年非小细胞肺癌协作组进行1, 394例NSCLC患者的荟萃分析，结果显示，与单纯手术组相比，手术加铂类为基础的化疗组5年生存率延长5%，虽然两组之间总生存期（overall survival, OS）并未达到统计学差异（HR=0.87, 95%CI: 0.74-1.02, *P*=0.08），但提示术后含铂辅助化疗可能有效^[13]^。2008年，LACECG荟萃分析纳入5项临床研究，共4, 584例NSCLC患者，中位随访时间为5.2年。结果发现，化疗组OS显著延长（HR=0.89，95%CI: 0.82-0.96, *P*=0.005），死亡风险下降11%，5年生存获益增加5.4%。相似的，化疗组无病生存期（diease free survival, DFS）显著延长（HR=0.84, 95%CI: 0.78-0.91, *P* < 0.001）^[9]^。而LACECG亚组分析显示，与观察组相比，行术后长春瑞滨/顺铂方案的Ⅲ期NSCLC患者获益最大（5年生存率提高14.7%），其次是Ⅱ期（5年生存率提高11.6%），Ⅰ期无显著获益（5年生存率提高1.8%）^[14]^。2010年NSCLCCG荟萃分析包括了26项随机研究，共纳入8, 447例NSCLC患者。结果发现，与单纯手术组相比，手术加化疗组5年生存率提高了4%（64% *vs* 60%, HR=0.86, 95%CI: 0.81-0.92, *P* < 0.001）^[15]^。2010年，我国的一项术后NSCLC辅助化疗的临床试验，包括了150例Ⅲa-N2 NSCLC患者，随机分为化疗组和观察组，中位随访时间为29个月。结果发现，化疗组OS和DFS均显著优于观察组（OS: HR=1.466, 95%CI: 1.017-2.114, *P*=0.037; DFS: HR=1.560, 95%CI: 1.064-2.287, *P*=0.037）^[16]^。

## 问题二：Ⅰ期-Ⅲa期行完全手术切除的NSCLC，若需要术后辅助治疗，需要何种类型的药物？

**推荐4：长春瑞滨/培美曲塞/紫杉醇/多西他赛/吉西他滨+铂类；1A**

2008年LACECG荟萃分析包括了5个含铂化疗方案的随机研究，共纳入4, 584例NSCLC患者，根据术后辅助化疗方案将所有患者分为两组，比较了顺铂/长春瑞滨和以铂类为基础的但不含长春瑞滨的化疗方案的优劣。结果显示，与其它化疗方案相比，顺铂/长春瑞滨化疗方案延长OS（HR=0.80, 95%CI: 0.70-0.91, *P* < 0.001, interaction *P*=0.04）和DFS（HR=0.75, 95%CI: 0.67-0.85, *P* < 0.001, interaction *P*=0.02）趋势更加明显^[9]^。相较于长春瑞滨/铂类方案，其他三代化疗药物为基础的方案在驱动基因阴性的晚期NSCLC治疗中疗效确切，专家一致推荐其用于Ⅰ-Ⅲa期完全术后NSCLC适宜人群的辅助化疗。

**推荐5：EGFR-TKI可用于Ⅲa期EGFR基因敏感突变患者；1B**

两项来自Memorial Sloan-Kettering Cancer Center（MSKCC）回顾性研究显示手术切除术后具有*EGFR*突变的Ⅰ期-Ⅲa期肺腺癌患者接受EGFR-TKI辅助治疗后DFS延长，复发风险降低，且OS有延长趋势^[17, 18]^。BR19研究是第一个随机、双盲、靶向药物用于完全切除Ⅰb期-Ⅲa期NSCLC辅助治疗的前瞻性研究，共纳入503例患者，按照1:1比例随机入组，安慰剂组252例，靶向治疗组251例，随访中位时间为4.7年，两组之间OS（HR=1.24, 95%CI: 0.94-1.64, *P*=0.14）和DFS（HR=1.22, 95%CI: 0.93-1.61, *P*=0.15）并无显著差异^[19]^。SELECT研究是一项在*EGFR*突变型NSCLC术后采用厄洛替尼辅助治疗的单臂、多中心、Ⅱ期研究^[20]^，该研究入组100例Ⅰa期-Ⅲa期NSCLC患者，中位随访时间为3.4年。结果显示服用厄洛替尼后2年DFS率达到了89%。RADIANT研究是迄今为止样本量最大的随机、双盲、Ⅲ期临床试验^[21]^，该研究纳入973例Ⅰb期-Ⅲa期NSCLC患者，按照2:1比例随机进入厄洛替尼组和安慰剂组，厄洛替尼组：厄洛替尼150 mg/d，治疗周期为2年。结果发现，厄洛替尼组和安慰剂组中位DFS分别为50.5个月和48.2个月，并未达到统计学差异（HR=0.90, 95%CI: 0.74-1.10, *P*=0.324）。而针对其中161例（16.5%）EGFR阳性的NSCLC患者进行亚组分析显示，与安慰剂组相比，厄洛替尼组中位DFS由28.5个月延长至46.4个月（HR=0.61, 95%CI: 0.38-0.98, *P*=0.039）。与安慰剂组相比，厄洛替尼组总生存期并无获益（HR=1.09, 95%CI: 0.55-2.16）。2017年ADJUVANT研究是第一个头对头比较术后靶向辅助治疗和术后辅助化疗的随机、开放、Ⅲ期研究^[22]^，该研究纳入222例Ⅱ期-Ⅲa期EGFR阳性（外显子19缺失或外显子21 L858 R突变）NSCLC患者，按照1:1比例随机进入靶向治疗组和化疗组，靶向治疗组：吉非替尼250 mg/d，治疗周期为2年；化疗组：长春瑞滨25 mg/m^2^，d1、d8，顺铂75 mg/m^2^，d1，每3周为1个周期，共4周期。中位随访时间为36.5个月。结果显示，与化疗组相比，靶向治疗组中位DFS由18.0个月延长至28.7个月（HR=0.60, 95%CI: 0.42-0.87, *P*=0.005, 4）。而且在安全性方面，吉非替尼组也优于化疗组。亚组分析显示DFS获益人群为N2期患者（HR=0.52, 95%CI: 0.34-0.80, *P*=0.003, 2），N1期并不能获益（HR=0.89, 95%CI: 0.45-1.76, *P*=0.743）。而EVAN研究是第一个比较厄洛替尼与化疗作为Ⅲa期*EGFR*突变NSCLC患者的辅助治疗疗效与安全性的多中心随机Ⅱ期研究^[23]^，该研究入组了102例NSCLC患者，按照1:1比例随机分组。厄洛替尼组：150 mg/d，治疗周期为2年；长春瑞滨/顺铂组：长春瑞滨25 mg/m^2^，d1、d8，顺铂75 mg/m^2^, d1，每3周为1个周期，共4周期。研究结果显示，与化疗组相比，厄洛替尼组疗效更优，2年DFS率显著提高（81.35% *vs* 44.62%, *P* < 0.001），中位DFS由21.0个月延长至42.4个月（HR=0.27, 95%CI: 0.14-0.53, *P* < 0.001）。而且厄洛替尼组安全性更好。OS数据虽然不成熟，但是厄洛替尼组OS具有获益趋势。ADJUVANT和EVAN研究开启了NSCLC辅助治疗的新纪元，两项研究均显示Ⅲa期*EGFR*突变NSCLC患者最有可能从术后辅助靶向治疗中获益，因此EGFR-TKI可作为Ⅲa期*EGFR*突变NSCLC患者完全切除术后的辅助治疗。

## 问题三：Ⅰ期-Ⅲa期行完全手术切除的NSCLC，哪些患者需要行术后辅助放疗?

**推荐6：Ⅰ期/Ⅱ期，不需要辅助放疗；1A**

1998年术后放疗荟萃分析试验组纳入了9项随机试验共2, 128例NSCLC患者，旨在对比单纯手术和手术联合术后放疗的疗效。其中Ⅰ期NSCLC患者562例，Ⅱ期718例。结果发现，与单纯手术组相比，手术联合术后放疗组死亡风险升高21%（HR=1.21, 95%CI: 1.08-1.34, *P*=0.001），2年生存率由55%下降到48%。手术联合术后放疗组2年无病生存率也下降4%（HR=1.13, 95%CI:1.02-1.26, *P*=0.018）。亚组分析显示这种不利影响主要体现在Ⅰ期/Ⅱ期NSCLC患者^[24]^。2005年该试验组更新了荟萃分析结果，纳入10项随机临床试验共计2, 232例NSCLC患者，但结果与之前报道一致，与单纯手术组相比，手术联合术后放疗组无论是OS（HR=1.18, *P*=0.002）还是DFS（HR=1.10, *P*=0.06）均差于单纯手术组。与前述研究一致，这种不利影响仍然主要体现在Ⅰ期/Ⅱ期NSCLC患者^[25]^。2013年该试验组再次更新了荟萃分析结果，纳入11项随机临床试验共计2, 343例NSCLC患者，但结果与之前报道一致，与单纯手术组相比，手术联合术后放疗组无论是OS（HR=1.18, 95%CI: 1.07-1.31, *P*=0.001）还是DFS（HR=1.10, 95%CI: 1.099-1.21, *P*=0.06）均差于单纯手术组。这种不利影响仍然主要体现在Ⅰ期/Ⅱ期NSCLC患者^[26]^。

**推荐7：Ⅲa期：不常规辅助放疗（术后综合评估辅助放疗对于每个N2期病人的效益和风险，当有证据支持，如多站淋巴结转移，考虑给予术后辅助放疗）；2A**

Corso等回顾性分析1998年-2006年美国国家癌症数据库（NCDB）中行R0切除的30, 552例Ⅱ期-Ⅲa期NSCLC患者，其中3, 430例患者接受术后放疗。研究发现与术后未接受放疗者相比，术后放疗降低了N0期（48% *vs* 37.7%, *P* < 0.001）和N1（39.4% *vs* 34.8%, *P* < 0.001）期患者的5年生存率。而N2期患者在接受术后放疗后5年生存率得到提高（27.8% *vs* 34.1%, *P* < 0.001）^[27]^。在辅助化疗成为Ⅲa-N2期NSCLC完全性切除术后标准治疗的前提下，Mikell等回顾性分析NCDB数据库中2004年-2006年间接受化疗的2, 115例N2期NSCLC患者，发现与对照组相比，术后放疗可提高5年生存率（39.8% *vs* 34.7%, *P*=0.048）^[28]^。同样地，Robinson等回顾性分析NCDB数据库2006年-2010年间接受化疗的4, 483例N2期NSCLC患者，结果同样显示术后放疗可显著提高5年生存率（39.3% *vs* 34.8%, *P*=0.014），中位生存也延长了4.5个月（45.2个月*vs* 40.7个月）^[29]^。Urban等对SEER数据库1998年-2009年行手术切除的11, 324例N1期-N2期NSCLC患者进行回顾性分析，其中N1期6, 551例（57.9%），N2期4, 773例（42.1%）。分析发现术后放疗可使N2期患者获益（HR=0.9, 95%CI: 0.84-0.99, *P*=0.026），而N1期患者并不能获益（HR=1.06, 95%CI: 0.97-1.15, *P*=0.2）^[30]^。Wei等^[31]^回顾性分析2004年-2013年SEER数据库中3, 334例行手术切除的Ⅲa-N2期NSCLC患者，研究结果显示，术后放疗能够提高总生存期（*P* < 0.001）。进一步分析发现生存获益主要集中在年龄小于60岁（5年生存率：35.4% *vs* 28.9%，*P*=0.026）和行肺叶切除（5年生存率：43.5% *vs* 34.5%，*P*=0.001）的NSCLC患者。2014年一项荟萃分析纳入11项Ⅲ期临床试验，共计2, 387例Ⅲa-N2期NSCLC患者，研究术后现代放疗技术在辅助治疗中的价值。结果显示：与对照组相比，无论是术后给予钴放疗、直线加速器放疗、还是二者联合均能够降低NSCLC局部复发。而只有当术后给予直线加速器放疗的时候才能提高总生存率（HR=0.76, 95%CI: 0.61-0.95, *P*=0.02）^[32]^。2013年术后放疗荟萃分析试验组再次更新了荟萃分析结果，纳入11项随机临床试验共计2, 343例NSCLC患者，但结果与之前报道一致^[24, 25]^，与单纯手术组相比，手术联合放疗组无论是OS（HR=1.18, 95%CI: 1.07-1.31, *P*=0.001）还是DFS（HR=1.10, 95%CI: 1.099-1.21, *P* =0.06）均差于单纯手术组。这种不利影响主要体现在Ⅰ期/Ⅱ期NSCLC患者。而对于Ⅲa期NSCLC患者，两组在总生存率和无病生存率方面并无明显差别，与单纯手术组相比，手术联合放疗组在局部复发率方面下降了24%^[26]^。

淋巴结站数同样影响术后辅助放疗的疗效。一项对Ⅲa-N2期完全切除术后的NSCLC患者辅助放疗的回顾性分析显示，单站N2亚组患者行PORT对比未做PORT组的生存无统计学差异；而多站N2患者中，PORT组的5年DFS率明显优于对照组（41.7% *vs* 5.9%, *P*=0.02）^[33]^。另外基于临床意见，对于右全肺切除患者，术后放疗需谨慎。

因此，术后辅助放疗可能提高Ⅲa-N2期NSCLC患者的生存，尤其对于多站淋巴结累及的患者获益明显。但是基于荟萃分析的结果，辅助放疗只是在局部复发率方面显出优势，在没有应用现代放射治疗手段的临床试验证明术后放疗确实可行之前，我们建议完全切除术后Ⅲa-N2期NSCLC不常规行术后辅助放疗。

## 总结及展望

随着精准治疗时代的到来，目前NSCLC的治疗更加精准，层出不穷的临床研究不断为其综合治疗增加循证医学的证据，使得NSCLC术后辅助治疗的获益人群更加精准，治疗方式更加合理，从而最终提高患者的生存率。当然，无论是系统治疗还是局部治疗，肿瘤分期都是需要考虑的关键因素。目前的证据显示：Ⅰ期患者术后不需要辅助化疗；Ⅱa期患者，综合考虑是否给予辅助全身治疗；对于Ⅱb-Ⅲa期行完全行切除的患者，推荐常规辅助药物治疗；Ⅰ期-Ⅲa期完全性切除的患者，术后不常规推荐辅助放疗，但对于部分N2患者，可酌情考虑辅助放疗；而对于存在*EGFR*敏感突变的Ⅲa期患者，术后推荐行EGFR-TKI治疗。另外还有问题仍存争议，值得关注：Ⅱa期高危因素人群的界定？N2期辅助放疗人群的选择与获益？Ⅲa期*EGFR*敏感突变靶向治疗的时长？Ⅱ期*EGFR*敏感突变的治疗选择？其他亚组人群如ALK融合阳性患者是否需要辅助治疗？抗血管生成治疗与免疫治疗在术后辅助治疗的定位？这些问题还需要积极探索，我们期待有更多的临床研究结果，尤其是国内的数据，来指导临床实践，最终转化为患者的生存获益（表 2）。

**2:** 汇总

序列	分期	推荐	级别	表述
问题一：Ⅰ期-Ⅲa期行完全手术切除的NSCLC，哪些患者需要行术后辅助治疗？				
推荐1	Ⅰ期^*^	不需要辅助化疗	1A	基于高水平证据，专家组统一共识，强烈推荐
推荐2	Ⅱ期^**^	不常规推荐辅助化疗 （术后综合评估包括与肿瘤内科专家会诊，评估辅助化疗对于每个病人的效益和风险，在做出建议时，还要考虑肿瘤分期以外的其他因素，包括组织病理学特征和基因改变等。当有证据支持，专家组有统一认识，利大于弊，可考虑给予辅助化疗）	2B	基于中等水平证据，专家组小有争议，一般推荐
推荐3	Ⅱb期-Ⅲa期	常规辅助化疗	1A	基于高水平证据，专家组统一共识，强烈推荐
问题二：Ⅰ期-Ⅲa期行完全手术切除的NSCLC，若需要术后辅助治疗，需要何种类型的药物？				
推荐4	Ⅱb期-Ⅲa期	长春瑞滨/培美曲塞/紫杉醇/多西他赛/吉西他滨+铂类	1A	基于高水平证据，专家组统一共识，强烈推荐
推荐5	Ⅲa期	EGFR-TKI可用于Ⅲa期EGFR基因敏感突变患者	1B	基于高水平证据，专家组小有争议，积极推荐
问题三：Ⅰ期-Ⅲa期行完全手术切除的NSCLC，哪些患者需要行术后辅助放疗?				
推荐6	Ⅰ期/Ⅱ期	不需要辅助放疗	1A	基于高水平证据，专家组统一共识，强烈推荐
推荐7	Ⅲa期	不常规辅助放疗 （术后综合评估辅助放疗对于每个N2期病人的效益和风险，当有证据支持，如多站淋巴结转移，考虑给予术后辅助放疗）	2A	基于中等水平证据，专家组统一共识，推荐
^*^Ⅰ期包括Ⅰa期，Ⅰ期，Ⅰa期：肿瘤直径≤3 cm，N0；Ⅰb期：肿瘤直径 > 3 cm，≤4 cm，N0；^**^Ⅱa期：肿瘤直径 > 4 cm，≤5 cm，N0.				

## 专家组成员

**发起人：**王长利（天津医科大学肿瘤医院）

**执笔：**岳东升（天津医科大学肿瘤医院），张华（天津医科大学肿瘤医院）

**参与专家（按姓氏笔画排序）：**马海涛（苏州大学附属第一医院），王群（复旦大学附属中山医院），支修益（北京宣武医院），毛伟敏（浙江省肿瘤医院），田辉（山东大学齐鲁医院），乔贵宾（广东省人民医院），刘永煜（辽宁省肿瘤医院），刘伟（吉林大学第一医院），刘伦旭（四川省华西医院），刘宏旭（辽宁省肿瘤医院），刘俊峰（河北医科大学第四医院），李小飞（唐都医院），李印（河南省肿瘤医院），李单青（北京协和医院），李强（四川省肿瘤医院），杨跃（北京肿瘤医院），汪栋（中国人民解放军第八一医院），张兰军（中山大学附属肿瘤医院），张百江（山东省肿瘤医院），张真发（天津医科大学肿瘤医院），张毅（北京宣武医院），陈海泉（复旦大学附属肿瘤医院），陈椿（福建医科大学附属协和医院），罗红鹤（中山大学附属第一医院），周清华（四川大学华西医院），赵珩（上海胸科医院），胡坚（浙江大学医学院一附院），徐世东（哈尔滨医科大学附属肿瘤医院），高树庚（中国医学科学院肿瘤医院），黄云超（云南省肿瘤医院），彭忠民（山东省立医院），蔡开灿（南方医科大学南方医院），魏煜程（青岛大学医学院附属医院）
